# The construction of patient quality management model in oral post anesthesia care unit in China: a grounded theory approach

**DOI:** 10.1186/s12871-023-02050-y

**Published:** 2023-03-28

**Authors:** Qi Zhang, Heshu Tang, Zhiqing Yao, Wei Han

**Affiliations:** 1 Nursing Department, Nanjing Stomatological Hospital, Medical School of Nanjing University, No. 30 Zhongyang Road, Nanjing, 210008 Jiangsu China; 2 Department of Urology, Jiangsu Province Hospital of Chinese Medicine, Affiliated Hospital of Nanjing University of Chinese Medicine, No. 155 Hanzhong Road, Nanjing, 210029 Jiangsu China; 3 Department of Oral and Maxillofacial Surgery, Nanjing Stomatological Hospital, Medical School of Nanjing University, No. 30 Zhongyang Road, Nanjing, 210008 China

**Keywords:** Grounded theory, Oral specialty, Post Anesthesia Care Unit, Patient quality management, Qualitative study

## Abstract

**Background:**

Nowadays, people have paid more and more attention to the quality of physical and mental health recovery after oral surgery anesthesia. As a remarkable feature of patient quality management, it can effectively reduce the risk of postoperative complications and pain in Post Anesthesia Care Unit (PACU). However, the patient management model in oral PACU remains unknown, especially in China. The purpose of this study is to explore the management elements of patient quality management in the oral PACU and to construct the management model.

**Methods:**

Strauss and Corbin’s grounded theory method was used to explore the experiences of three anesthesiologists, six anesthesia nurses and three administrators working in oral PACU. Twelve semi-structured interviews were conducted using face-to-face in a tertiary stomatological hospital from March to June, 2022. The interviews were transcribed and thematically analysed according to QSR NVivo 12.0 qualitative analysis tool.

**Results:**

Three themes and ten subthemes were identified through an active analysis process, including three of the core team members: stomatological anesthesiologists, stomatological anesthesia nurses and administrators, three of the main functions: education and training, patient care and quality control and four of the team operation processes: analysis, plan, do, check.

**Conclusion:**

The patient quality management model of the oral PACU is helpful for the professional identity and career development of stomatological anesthesia staff in China, which can accelerate the professional development of oral anesthesia nursing quality. According to the model, the patient’s pain and fear will decrease, meanwhile, safety and comfort will increase. It can make contributions to the theoretical research and clinical practice in the future.

## Background

With the expansion of the scope of modern oral and maxillofacial surgery and the complex oral surgery procedures, the oral Post Anesthesia Care Unit (PACU) plays an increasingly important role now. Oral surgery procedures are often perceived to be difficult resulting in onward referrals to secondary care either due to diminished remuneration, lack of confidence in undertaking surgical procedures or managing complex medical comorbidities [1]. Oral and maxillofacial surgery involves a wide range of operations, so patients are often given general anesthesia support to complete the operation. However, patients’ bodies are affected by the nerve block effect caused by anesthetic drugs and surgical stress reaction, so they need to stay in PACU for monitoring after surgery and can leave the room until they regain their normal consciousness [2]. PACU is a special anesthesia nursing unit for postoperative patient resuscitation monitoring and treatment, which has the characteristics of large patient flow, fast turnover and high risk of illness [3]. As we enter the third winter of Covid-19 pandemic, no one could have anticipated the challenges that COVID-19 has wrought on every aspect of our lives, both personally and professionally, adding to already increased pressures in primary care and compounding the burden on increasingly large waiting lists for treatment on referral [4]. The continuous growth of operation volume has brought great pressure to patient quality management in oral PACU. To ensure the safety and quality of life outcome of patients is important factor to construct a standardized patient quality management model.

In China, all stomatological hospitals follow the guidelines of general hospitals for the management of patient quality in oral PACU, but in fact, the management in oral PACU not only has great differences in nursing observation and medical treatment, but also lacks a scientific theoretical model. Less attention was paid to the patient quality management of PACU in stomatological hospitals. In order to truly reflect the necessity of establishing the oral patient management model, our research group have investigated the data of PACU patients from March to June, 2022 in Nanjing Stomatological Hospital. We found that the common types of surgery in our hospital are jaw fracture surgery, oral cancer surgery, orthognathic surgery, extraction of impacted teeth under general anesthesia, jaw cyst removal surgery and parotid gland surgery. Additionally, there were 1201 patients in oral PACU and 210 are children involved. Most children patients mainly underwent tooth extraction surgery and 338 are geriatric patients mainly underwent oral cancer and maxillofacial fracture surgery. The proportion of patients under anesthesia I ~ II in ASA classification is 95.28%. During this period, the complications easily occurred and the incidence rate was about 0.017% ~ 17.37% in our oral PACU. The most common complications are pain and dysphoria (n = 319), difficult airway(n = 72) and delayed recovery(n = 6). The notable difference between general hospitals and stomatological hospitals is that we need to pay more attention to the observation of respiratory tract, especially in caring for geriatric patients and children.

Therefore, this study deeply explored the working environment of oral PACU medical staff in terms of job responsibilities, work flow, work cooperation, etc. It is the first qualitative study to explore the construction elements of the patient quality management model in the oral PACU, which provides theoretical guidance for the construction of the management model in the oral PACU.

## Methods

### Study design and setting

Strauss and Corbin’s grounded theory method [5] was conducted in the form of semi-structured interviews with the medical staff of the stomatological hospital, Nanjing, Jiangsu Province and adhered to the COREQ guidelines. This grounded method emphasizes that concepts, abstract categories, build associations, summarize and refine the actual objects and direct investigation data through continuous comparison and theoretical sampling, so that new theories emerge naturally from bottom to top [6]. Therefore, based on literature review and comprehensive consideration of the extensive application in academic circles, this study employs this grounded theory to build a patient quality management model in oral PACU.

### Participants

The study was conducted from March to June, 2022 and adopted purposeful sampling method in order to effectively analyze typical cases and dig deep into the experience of the research object [7, 8]. The medical staff (n = 12) of a tertiary stomatological hospital in Nanjing, Jiangsu Province were selected as the interviewees. Inclusion criteria: (1) Bachelor degree or above; (2) Having 5 years or more working experience in clinical/nursing work of oral PACU; (3) Having a certain understanding of this study. The researchers explained the purpose and significance of the interview to the participants, and the respondents signed informed consent forms. The real names of respondents are replaced by N1-N12.

### Ethical considerations

The protocol was approved (Approval Number NJSH-2021NL-108) on 21 December 2021 by the Ethics Committee of Nanjing Stomatological Hospital (Medical School, Nanjing University, Jiangsu, China). Written informed consent was obtained from all participants according to the local regulations and to the principles of Helsinki Declaration. Prior to the interviews being conducted, participants were informed about the aim of the study and provided written informed consent. However, participants were informed that they could withdraw at any time from the study without any negative consequences. All data were confidential. Participants’ data were accessible only to the researchers who involved in the study and all document files were eradicated immediately following data analysis.

### Data collection procedures

A semi-structured interview guide was developed collaboratively by research team. According to theoretical knowledge, literature research results, professional knowledge and clinical experience, we created open-ended questions. A pre-interview was conducted before the formal interview, and the interview outline was adjusted according to the pre-interview content and discussion. The final interview content was composed of four open questions. To get the informants to elaborate on their experiences, we used words, such as: ‘Tell, describe, what do you think, how did you perceive, and what happened then?’. In-depth interviews were conducted with participants, who were asked the following questions:

1. What do you think are the core personnel to build the management model of patient quality in oral PACU?
2. What do you think are the main responsibilities of patient quality management in oral PACU at present?
3. What do you think are good or bad of patient quality management? Please elaborate on the reasons.
4. In your opinion, what aspects should be improved in the management in oral PACU?

According to the interviewee’s preference, a semi-structured interview was applied to collect data through face-to-face interviews. The interview was held in a relaxed and comfortable setting, such as the interviewees’ office. Two nursing postgraduates with oral anesthesia nursing knowledge and qualitative research training asked questions, digitally audio-recorded and were transcribed verbatim with participants’ permission. Meanwhile, the interviewer recorded the facial expressions, special emotions, pronunciation and body movement changes of the interviewees during the interview and wrote a memorandum [8]. The interviews were conducted until thematic saturation was reached (no new themes and patterns emerged), with confirmation by the principal investigator (RS). After the interview, the interviewer wrote a reflection diary. Finally, all the 12 interviewees agreed to participate in this study and no one quitted. The code in turn according to the interview sequence N (1, 2, 3…) converted the recorded files into words after double check within 24 h, discussed the controversial parts in groups, verified the interview contents with the interviewees, and entered them into NVivo12.0 software after no objection.

### Statistical analysis

**Fig. 1:**
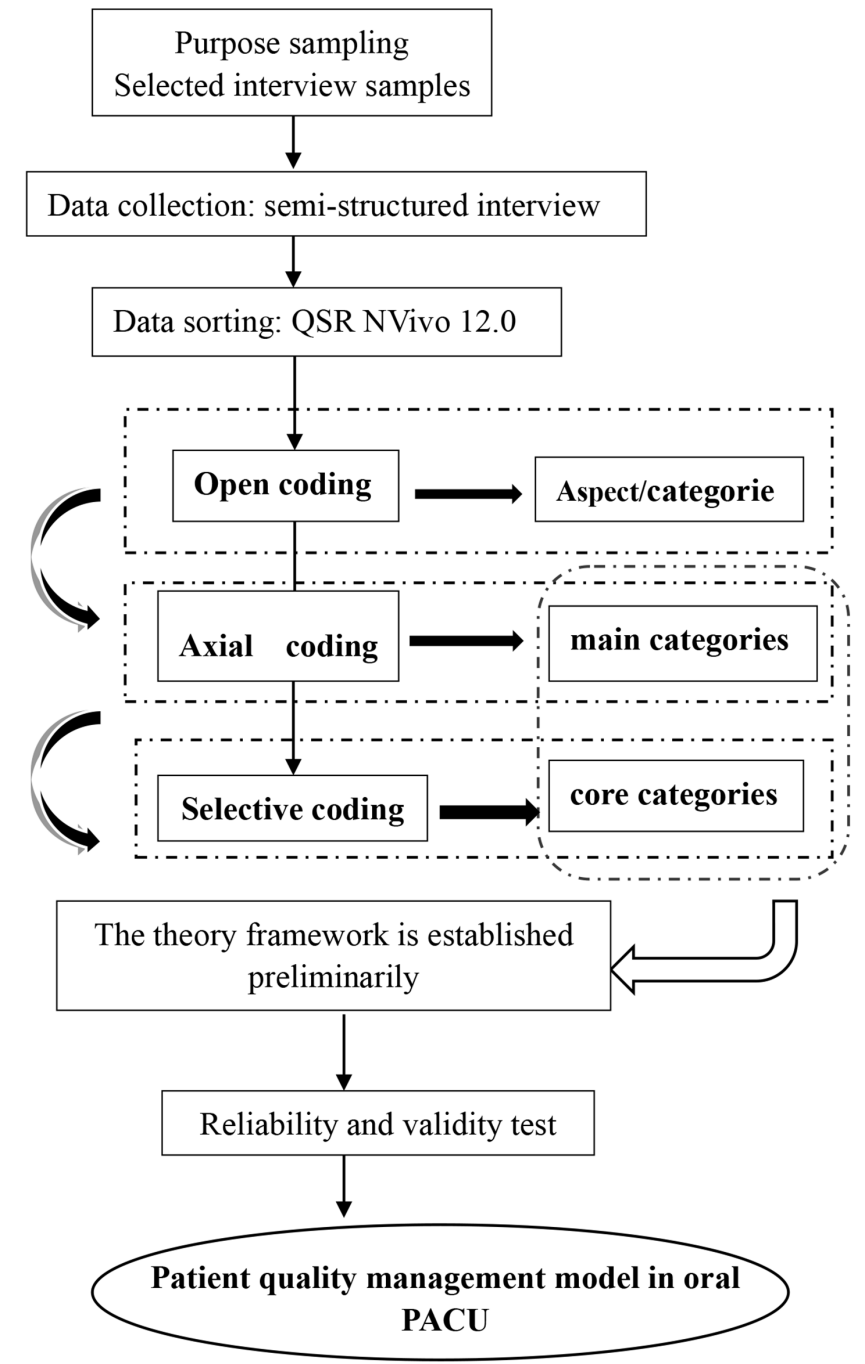
Data collection and analysis process

According to Corbin and Strauss’s theory, the transcripts from the interview were analysed based on the following coding procedures: (1) open coding, (2) axial.

coding and (3) selective coding. At the open coding stage, the transcripts were conceptualized and then clustered into subcategories during the coding stage. Researchers analysed the subcategories and explored the relationship to develop main categories at the axial coding stage. And at the selective coding stage, the interrelationship among main categories had been further interpreted to form the core categories. To ensure the core categories can explain all of the main categories, they should be closely related [9, 10]. To avoid bias and achieve greater precision during the coding process, constant comparisons were used while analysing the data. To improve trustworthiness and credibility, the survey data were coded and constantly compared with the data from the interview after homogeneous categorization. Furthermore, before coding the survey descriptions, they were screened against the following criteria:(1) The descriptions were clear (2) the leaders’ behaviors or traits were demonstrated and (3) the descriptions were pertinent to the topic. The first and second authors completed this process and integrated similar descriptions for further analysis. Researchers repeatedly read the transcripts and descriptions to ensure their familiarity and sensitivity to the data in case of missing important information. The authors encoded the original transcripts and descriptions separately during the coding stage and then compared the results until the codes were consistent. If there were coding disagreements, seek assistance from the corresponding author to make a final decision [11]. QSR NVivo 12.0 software was used to analyse the transcripts and integrated descriptions. Figure 1 presents the data collection and analysis process.

## Results

An individual in-depth interview lasted about 20-45 mins and the whole process was recorded. Table 1 demonstrates the demographic characteristic of all participants(N1-12).

**Table 1:** the demographic characteristic of all participants(N1-12)

No.	Gender	age(year)	Degree	Areas of expertise	Years of work (year)	Position
N1	male	56	M.M	Oral anesthesia	25	Administrator
N2	male	49	M.M	Oral anesthesia	24	Anesthesiologist
N3	male	34	M.D	Oral anesthesia	10	Anesthesiologist
N4	male	27	M.M	Oral anesthesia	5	Anesthesiologist
N5	male	26	B.N	Anesthesia nursing	5	Anesthesia nurse
N6	female	45	B.N	Nursing management	24	Administrator
N7	female	38	B.N	Anesthesia nursing	20	Administrator
N8	female	36	B.N	Anesthesia nursing	16	Anesthesia nurse
N9	female	32	B.N	Anesthesia nursing	10	Anesthesia nurse
N10	female	26	B.N	Anesthesia nursing	6	Anesthesia nurse
N11	female	33	B.N	Clinical nursing	12	Anesthesia nurse
N12	female	26	B.N	Clinical nursing	5	Anesthesia nurse

**Fig. 2:**
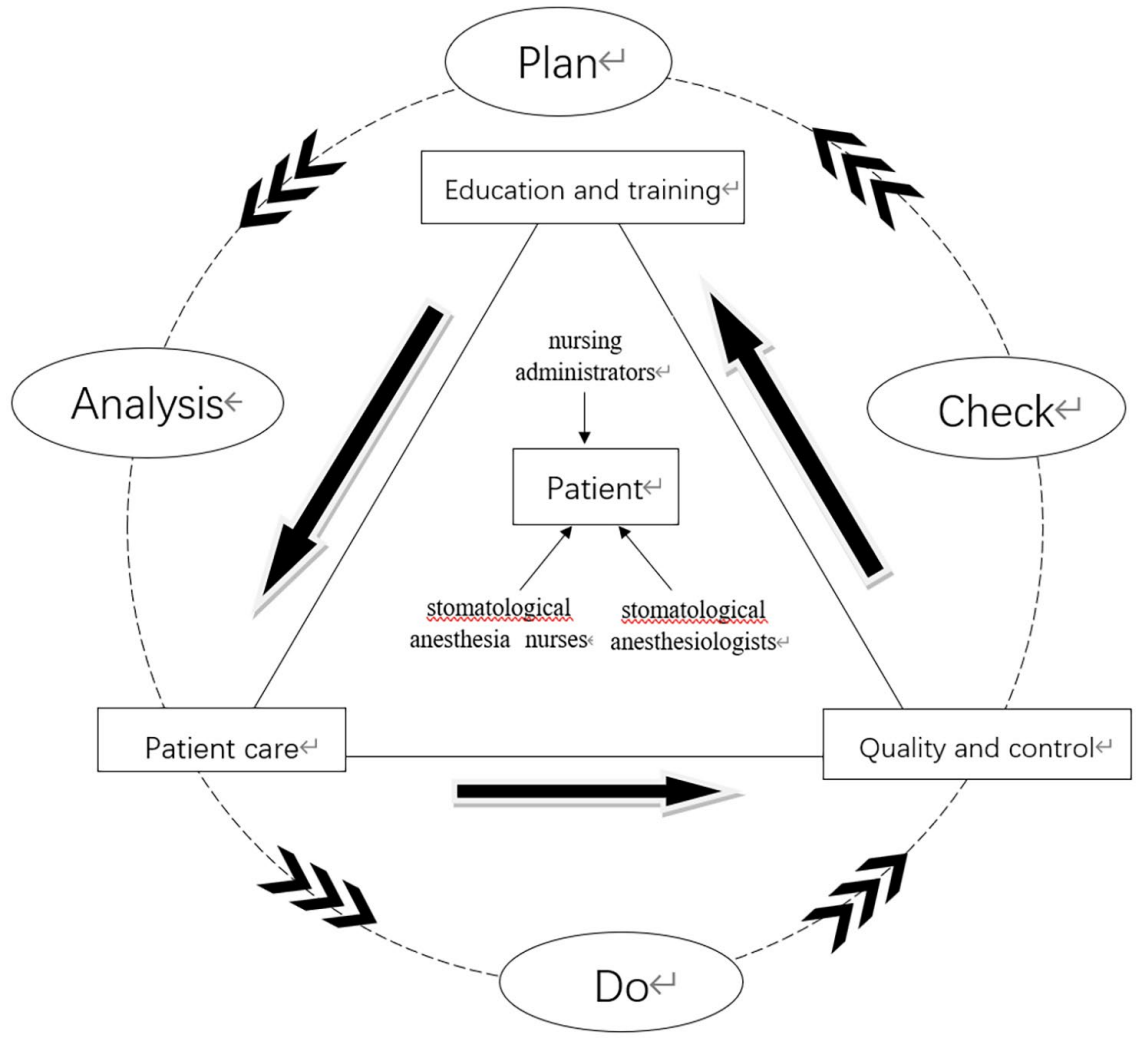
Patient quality management model

The framework of the patient quality management model emerged as a result of multiple levels of coding and constant comparison. The coding process is shown in Table 2 (attached at the end of the document text file). The model represented the core elements of patient management in oral PACU, how this model works and possible influence of patient management, present in Fig. 2.

**Table 2:** Coding process of patient quality management model

Selective coding	Axial coding	Open coding
three of the core team members	stomatological anesthesiologists	Guidance role
		Provide technical support
		Good cooperator
	stomatological anesthesia nurses	The core force
		Professional qualification
	nursing administrators	No specialized leader
		The shift arrangement of anesthesia nurses is not standardized
		Unclear management process
Three of the main functions of the team	Education and training	Oral surgery knowledge training
		Emergency dyspnea training
		Head and neck wound risk identification
		PACU handover system learning
	Patient care	Humanistic care for reviving fear
		Airway management
		Hypothermia management
		Respiratory tube protection
		Deep vein, peripheral vein and artery protection
		Management of anesthetic drugs
	Quality control	Consciousness score of transferred out patients
		Management of patients with delayed resuscitation
		Pain evaluation of transferred out patients
		Monitoring of ECG monitoring instruments and equipment
		Unclear Transferred in handover items
Four of the team operation processes	Analysis	Observation of respiratory conditions of patients
		Large age span of patients in stomatology department
		Difficulty in comprehensive monitoring without centralized monitoring display
	Plan	Communication and coordination
		Key management plan for postoperative patients
		Standardized nursing operation process
	Do	Strengthen the training of maxillofacial surgery knowledge
		Weekly group meeting to report the learning situation
		Do not only shout slogans but also develop a reward mechanism
	Check	Focus on the key indicators of patients in the recovery period of anesthesia
		Anonymous feedback box of organization team
		Establishment of risk early warning system

## Discussion

### From the perspective of nursing administrators to carry out education and training

As the “leader” of the team and the “commander” of education and training, nursing administrators play a role in the team from point to area. Compared with basic anesthesia nursing education, continuing education pays attention to the cultivation of critical thinking of anesthesia nursing workers, emphasizes the deep combination of knowledge and clinical cases and pays special attention to the treatment of complex and difficult cases in work [12]. This model suggests that nursing administrators should regularly carry out oral disease knowledge training related to the stomatological anesthesia nurses in a planned and directional way. As one member said:

In addition to specialist nursing, such as oral diseases, the possible sudden complications need to be understood more. If she doesn’t even know this, then the critical attention will not be found, and there is no way to correctly judge the situation of each patient

A common theme for the anaesthesia personnel was that they learned a lot about different medications and diseases, which they considered important knowledge [13].

It is helpful to improve the professional skills and professional identity of stomatological anesthesia nurses in strengthening airway management, risk identification related to general anesthesia and monitoring training in patient circulation.

### From the perspective of stomatological anesthesia nurses to lead patient care

As the core strength of patient quality management team in oral PACU, anesthesia nurses play the role of main force in the team and control the management process. Anesthesia nurses are the main duty bearers of the oral PACU. Anesthesia nurses need to manage patients from the time when patients enter PACU to the time when patients are not awake to awake and then leave. The core concept of nursing is “patient-centered”. The turnover of patients in oral PACU is generally 1.5-2 h and the patient’s condition change rapidly, which requires higher nursing quality. In the nursing process, there are differences between anesthesia nursing and routine disease nursing [14]. As one member mentioned:

Because each patient has different characteristics, for example, patients with parotid gland, orthognathic jaw or malignant tumors. We need to know the knowledge and characteristics of oral diseases in addition to specialist operations.

It is necessary to strengthen the pipeline management of anesthesia nurses for patients, such as respiratory tract, deep vein, peripheral vein and artery. Then, as one of the five basic patient indicators, patients are more likely to have hypothermia symptoms after anesthesia. Oral anesthesia nurses should further strengthen the management of basic vital signs of patients such as the improvement of hypothermia symptoms and the improvement of pain evaluation process. By clarifying nursing responsibilities and refining the key points of nursing measures, the nursing quality can be improved and professional identity of anesthesia nurses can be further improved.

### From the perspective of stomatological anesthesiologists to improve quality control

The construction of patient management team focuses on cooperation with team members. Undoubtedly, anesthesiologists play an important guiding role in the team. As nursing collaborators, anesthesiologists can provide theoretical guidance and technical support for anesthesia nurses, participate in the discussion of difficult cases and give corresponding countermeasures. At the same time, anesthesiologists play an important role in quality control. As one member said:

Anesthesia nursing work is mostly directed by anesthesiologists. all invasive operations should be under the guidance of anesthesiologists and close cooperation with anesthesiologists.

“E Guanzi Tianze” said: “One leaf covers one’s eyes, but no mountain is seen”. Oral anesthesiologists can give constructive professional suggestions in the field of stomatology, guide the nursing operation of anesthesia nurses and give feedback and guidance to anesthesia nurses in time to better improve the patient quality of PACU. Moreover, the oral anesthesiologist has played a key role in the pain management of patients. The anaesthesia personnel work together to do a good job in the pain management of patients during the recovery period after anesthesia, so that patients can reduce the pain and discomfort after anesthesia. Therefore, it is a necessary step that oral anesthesiologists can control patient management quality.

### Efficient operation of oral PACU patient quality management model

The operation process in oral PACU follows PDCA cycle theory model, including analysing problems (Analysis), making plans (Plan), training implementation (Do) and quality control (Check). By forming a Deming ring cycle closed quality control circle, it provides a specific executable management method, Stomatological anesthesiologists and anesthesia nurses found clinical patient management problems in PACU. They formed a quality control circle group with nursing administrators and formulated plans to improve patient quality by clarifying their respective responsibilities. Through continuing education and training assessment for anesthesia nurses, the anesthesia nursing level of stomatology specialty is linked with the quality of patients, and finally developed and improved in a spiral way to realize the efficient operation of the team. In the future, we plan to implement this management model to evaluate the effect of the six aspects of the patient’s restlessness(Richmond Agitation-Sedation Scale, RAS) during the awakening period, the patient’s pain management evaluation(visual analogue scale rule ,VAS), the incidence of complications, the retention time of PACU, the patient’s satisfaction, and the satisfaction of medical staff. And our model will constantly improve the quality according to the feedback.

## Conclusions

This study is the first qualitative study to explore the perspectives of medical staff in oral PACU about construction of the patient quality management model in China. The study is based on the qualitative research method of grounded theory, in-depth interviews were conducted with medical staff in PACU of the stomatological hospital. By scientifically standardizing the three-stage coding program, summarize and refine the empirical model from the interview materials. Finally, we constructed the patient quality management model in oral PACU.

However, the data collection of this study is limited to a stomatological hospital in Nanjing, Jiangsu Province. And does not extensively involve other relevant personnel in the team building practice. It is necessary to expand the research population and research areas. We hope further carry out multi-center and multi-angle research to focus on the construction of professional patient quality management teams and continue to improve the theoretical model in combination with quantitative research.

## Data Availability

The datasets used and/or analysed during the current study are available from the corresponding author on reasonable request.

## List of abbreviations

PACU: Post Anesthesia Care Unit
COREQ: Consolidates Criteria for Reporting Qualitative Research
M.M: Master of Medicine
M.D: Doctor of Medicine
B.N: Bachelor of Nursing
PDCA: P—plan, D—do, C—check, A-Analysis

## References

[1] Wilson N. Review of oral surgery services and training. In: London: Medical Education England. 2010. https://www.baos.org.uk/resources/MEEOSreview.pdf. Accessed Jun 2022.

[2] Jiang AG, Liu DF, Fan XL (2018). Effect of comprehensive dental care on the postoperative quality of life in patients undergoing oral and maxillofacial surgery [J]. China Rural Health Administration.

[3] Weissman C, Scemama J, Weiss YG (2019). The ratio of PACU length-of-stay to surgical duration: practical observations. Acta Anaesthesiol Scand.

[4] Shah A (2022). Oral surgery. Prim Dent J.

[5] Strauss A, Corbin JM. Grounded theory in practice. Sage; 1997.

[6] Barney G, Glaser AL, Strauss (1967). The Discovery of grounded theory: strategies for qualitative research.

[7] Smith JA (1996). Beyond the divide between cognition and discourse: using interpretative phenomenological analysis in health psychology. Psychol Health.

[8] Smith JA (2011). Evaluating the contribution of interpretative phenomenological analysis. Health Psychol Rev.

[9] Corbin JM, Strauss A (1990). Grounded theory research: procedures, canons, and evaluative criteria. Qualitative Sociol.

[10] Kempster S, Parry KW. Grounded theory and leadership research: A critical realist perspective.The Leadership Quarterly.2011.10.1016/j.leaqua.2010.12.010.

[11] Zhang F, Peng X, Huang L (2022). A caring leadership model in nursing: a grounded theory approach. J Nurs Manag.

[12] Chen SR, Zhang HM, Zhi H (2022). Research progress of application in continuing education and training of anesthesia nurses based on the application of complex adaptive system theory. J Nurses Train.

[13] Kristoffersen EW, Opsal A, Tveit TO (2022). Knowledge, safety, and teamwork: a qualitative study on the experiences of anaesthesiologists and nurse anaesthetists working in the preanaesthesia assessment clinic. BMC Anesthesiol.

[14] Vacheron CH, Peyrouset O, Incagnoli P (2021). Multitasking in postanesthesia care unit following nurse interruptions, an analysis of the causes and consequences using classification tree: an observational prospective study [published online ahead of print, 2021 Jun 9]. Braz J Anesthesiol.

